# Accounting for tourism benefits in marine reserve design

**DOI:** 10.1371/journal.pone.0190187

**Published:** 2017-12-21

**Authors:** Daniel F. Viana, Benjamin S. Halpern, Steven D. Gaines

**Affiliations:** 1 Bren School of Environmental Science & Management, University of California, Santa Barbara, Santa Barbara, California, United States of America; 2 National Center for Ecological Analysis and Synthesis, University of California, Santa Barbara, Santa Barbara, California, United States of America; 3 Silwood Park, Imperial College London, Ascot, United Kingdom; Sveriges lantbruksuniversitet, SWEDEN

## Abstract

Marine reserve design often considers potential benefits to conservation and/or fisheries but typically ignores potential revenues generated through tourism. Since tourism can be the main source of economic benefits for many marine reserves worldwide, ignoring tourism objectives in the design process might lead to sub-optimal outcomes. To incorporate tourism benefits into marine reserve design, we develop a bioeconomic model that tracks tourism and fisheries revenues through time for different management options and location characteristics. Results from the model show that accounting for tourism benefits will ultimately motivate greater ocean protection. Our findings demonstrate that marine reserves are part of the optimal economic solution even in situations with optimal fisheries management and low tourism value relative to fisheries. The extent of optimal protection depends on specific location characteristics, such as tourism potential and other local amenities, and the species recreational divers care about. Additionally, as tourism value increases, optimal reserve area also increases. Finally, we demonstrate how tradeoffs between the two services depend on location attributes and management of the fishery outside marine reserve borders. Understanding when unavoidable tradeoffs will arise helps identify those situations where communities must choose between competing interests.

## Introduction

Degradation of ocean ecosystems driven by human activities has led to an increased global interest in the establishment of ocean protected areas [1,2]. One type of protected area, where all forms of fishing are prohibited, is known as a “marine reserve” [3]. Much of the interest in marine reserves is driven by their success in recovering important habitats and increasing species biomass and diversity within the reserve’s boundaries [4]. Although reserves can fail to reach their full potential because of the lack of resources for monitoring and enforcement [5], they are a globally important conservation tool. In addition to these clear conservation benefits, the increases in species population size within reserves can also generate important economic benefits. For example, fisheries benefits can arise through the spillover of adults and/or the export of larvae to surrounding fished areas [6,7].

Several frameworks have now been developed to help capture these joint conservation and economic benefits in effective marine reserve designs [8–10]. One key limitation of the existing work, however, is that by focusing primarily on fisheries economic benefits it has ignored a potentially far larger source of added revenues—tourism. Tourism gains can be obtained through diving operations within the marine reserve [11,12] and the consequent multiplier effects on local businesses related to tourism (e.g. hotels, restaurants). Collectively, these tourism benefits can be the main source of economic gains from many marine reserves worldwide [13,14]. To date, there is no clear framework to maximize these potential benefits through effective reserve design. As a result, key questions remain, such as: will the range of conditions where marine reserves are profitable conservation tools grow when tourism is accounted for, and are there inherent economic tradeoffs between reserve benefits to fisheries versus tourism?

Marine reserve benefits to conservation, fisheries and tourism all depend on the buildup of biomass and diversity of species within their borders. Thus, many design elements (such as appropriate reserve size relative to scales of fish movement) might align regardless of reserve objectives, while others might be at odds with each other. For example, while fisheries benefits depend on the spillover of adults and/or larval export, tourism and conservation benefits may benefit from higher levels of local retention. This can have important implications in terms of edge location and size of the reserve [3]. Additionally, optimal location of a marine reserve in relation to the coast might differ depending on the objective. Placing a reserve close to port may decrease costs for tourism operators and enforcement agencies while at the same time increase costs for fishers, since they will have to travel longer distances to reach their fishing grounds. Moreover, while conservation objectives require protection of all threatened species and habitats, reserves designed for tourism or fisheries objectives might require only protection of some key species and habitats. This distinction can have important design implications in relation to the location and size of reserves [15].

Studies have shown that divers and snorkelers consider ecosystem characteristics and other local amenities when deciding where to visit [16]. Divers are attracted to conservation gains of marine reserves [17] such as increases in the abundance of fish, the diversity of species, iconic species, and coral reef conditions [18–20]. Additionally, since divers are also tourists, other local amenities can also play an important role. Characteristics such as tourism infrastructure, local attractions, proximity to airports, and quality of restaurants and hotels can directly influence a diver’s decision on where to visit [21]. Relative importance of local amenities versus ecosystem health depends on divers’ preferences and availability of different habitats and species. For example, in the Great Barrier Reef, Australia, whales and dolphins were the preferred draws for divers followed by sharks and rays, overall species richness, turtles and large fish [22]. In the western Caribbean islands, variety of fish, fish abundance and coral variety were the preferred attributes [19]. In contrast, divers from Barbados listed terrestrial characteristics (beaches) and warm and clear water as their main reason for visiting the area followed by coral and fish diversity and abundance [23]. Such differences in preferences show evidence of two categories of divers, one category that is driven by ocean biodiversity and another category that is mainly driven by other local amenities [23]. The former group will likely be attracted by marine reserves, while the latter may be indifferent.

Benefits from tourism can in many cases be far greater than the opportunity cost of foregone fishing. For example, in the Great Barrier Reef annual revenue from tourism is 36 times greater than income from commercial fishing [14]. In the Medes Islands Marine Reserve (Spain) annual revenue from tourism is about 20 times greater than fishing revenue [24]. Potential tourism revenue from marine reserves can be generated directly through user fees [12] or by boosting the tourism economy in the region. Marine Reserves can potentially increase value of all business associated with tourism (e.g. hotels, restaurants), especially those dependent on underwater activities (e.g. dive centers). These benefits depend on the location of the reserve as well as the biomass of fish in the water [16]. Reserves located near coastal areas with intense tourism activity and other tourist attractions are likely to have high visitation rates quickly after reserve creation [25]. In such situations, the marine reserve may not be the main draw to the area and often does not require high levels of biomass to attract divers. By contrast, locations where there are no other coastal attractions other than the marine reserve may only attract more experienced divers that are drawn by high levels of fish biomass and diversity [26]. These areas may need to be more spectacular and tied with marketing strategies to attract large numbers of divers, since the reserves will often be competing with diverse diving options around the globe.

Despite growing evidence of economic benefits associated with tourism activities in marine reserves, most spatial planning models only take into account fisheries and/or conservation benefits but ignore tourism gains. To incorporate potential tourism benefits we develop a bioeconomic model to simulate different marine reserve designs and their predicted impacts on fisheries and tourism revenue. We model the potential benefits for both services under different tourism and fisheries management scenarios to ask under which conditions are marine reserves part of the optimal solution that maximizes total economic benefits. We then analyze the potential tradeoffs between fisheries and tourism economic benefits to understand the incentives stakeholders face and the situations where conflicts are likely to arise.

## Material and methods

We use a bioeconomic model to simulate different marine reserve designs and the potential economic benefits to fisheries and tourism over time. We divide a hypothetical coastline into 100 homogeneous linear patches where we track the biomass within each patch. Patches are wrapped to eliminate any boundary effect and to make sure all patches are homogeneous. Patches are connected through adult spillover. A fraction of the population emigrates from each patch to nearby patches with a probability that depends on the distance between the patches. A certain fraction of the biomass is also removed through fishing from each patch that is not a marine reserve, with the sum of discounted revenues over time representing the economic gains to fisheries. Larval dispersal is assumed to occur within each patch as population growth in a patch is only dependent on local population size. Although we acknowledge the important design implications driven by larval dispersal dynamics [27,28], we did not consider larval connectivity to simplify the model. Tourism benefits are associated with an increase in the demand for dives inside the marine reserve associated with increased fish density [16]. We did not consider diving activities in fished areas since our source of revenues are the user fees charged to gain access to the marine reserves.

### Biological model

We use a simple logistic model that tracks biomass of a given species in each patch over time:

$$B_{t,i}=B_{t-1,i}+g{*B}_{t-1,i}*\left(1-\frac{B_{t-1,i}}{K_{i}}\right)-f_{i}*B_{t-1,i}-E_{t,i}+I_{t,i}$$ (1)

Where *B*_*t*,*i*_ is the biomass in year *t* and patch *i*, *g* is the intrinsic growth rate, *K*_*i*_ is the carrying capacity, *f*_*i*_ is the harvest fraction, *E*_*t*,*i*_ is the emigration from patch *i* and *I*_*t*,*i*_ is the immigration to patch *i* from all other patches.

Harvest fraction in each patch, *f*_*i*_, is calculated according to Hilborn et al. 2006, where the intensity of harvest is proportional to the biomass in each patch. We assume that total effort remains constant when a marine reserve is created. This translates to an increased fishing intensity in areas open to fishing as the size of marine reserves grows. The combination of a constant overall fishing effort and a resulting fixed fishing mortality rate in fished patches accounts for the displacement of effort caused by marine reserve placement and creates the fishing the line effect [29] associated with higher catches in patches surrounding marine reserves. For well managed scenarios, total fishing effort is calculated as the amount that generates maximum sustainable yield at equilibrium when the entire area is open to fishing. For overfished scenarios, we assume a fishing effort that would drive fish biomass down to 10% of carrying capacity at equilibrium when all patches are open to fishing. This open access equilibrium biomass value was assumed according to [30]. Harvest fraction inside patches designated as marine reserves is zero. Initial biomass is assumed to be the equilibrium biomass under the different fisheries management scenarios (50% and 10% of carrying capacity for well managed vs. overfished, respectively).

Emigration from patch *i* (*E*_*i*_) equals the biomass of fish in the previous year, B_t-1,i_, times the movement fraction, represented by μ:

$$E_{t,i}=B_{t-1,i}*\mu$$ (2)

Immigration to patch *i* (*I*_*i*_) is the sum of the emigration contributions from all other patches *j*:

$$I_{i,t}=\sum_{j\;=1}^{100}E_{j,t}p_{ji}$$ (3)

where the proportion of emigrant fish moving from each patch *j* to patch *i*, *p*_*ji*_ is defined as [16]:

$$p_{ji}=exp(-d_{ji})$$ (4)

where *d*_i,j_ is the distance between patch *j* and patch *i*. Relative proportions are then normalized so that the proportions moving to all other patches sum to one.

### Economic model

#### Fisheries value

Fisheries revenue (*R*_*t*_) is the sum across all patches of the product of the harvest fraction (*f*_*j*_), resource price (λ) and biomass (B_t,i_) in year *t*.

$$R_{t}=\sum_{j=1}^{100}f_{j}*B_{j}*\lambda$$ (5)

Total net present value of fisheries revenue (FV) is then calculated by summing across all years and applying a discount rate:

$$FV=\sum_{t=1}^{50}R_{t}*{\left(\frac{1}{1+\delta }\right)}^{t}$$ (6)

Where *δ* is the discount rate.

#### Tourism value

Tourism value is assumed to be associated with the density of fish inside the marine reserve to reflect the underwater experience of divers. As described by Sala et al. 2013, we assume a dive’s marginal value is directly influenced by the diver’s underwater experience. Increased fish density inside the marine reserve will shift diver’s demand outward, increasing potential revenue generated from the system [16]. Additionally, we assume a congestion effect restricting the total number of divers per marine reserve area per unit time. This reflects the fact that divers prefer less crowded areas, and marine reserves often adopt a cap on the total number of dives per day per area of marine reserve to ensure conservation benefits. Such policy results in a diver carrying capacity inside the marine reserve. The size of the reserve thus limits the potential number of dives per marine reserve area per unit time.

**Fig 1:**
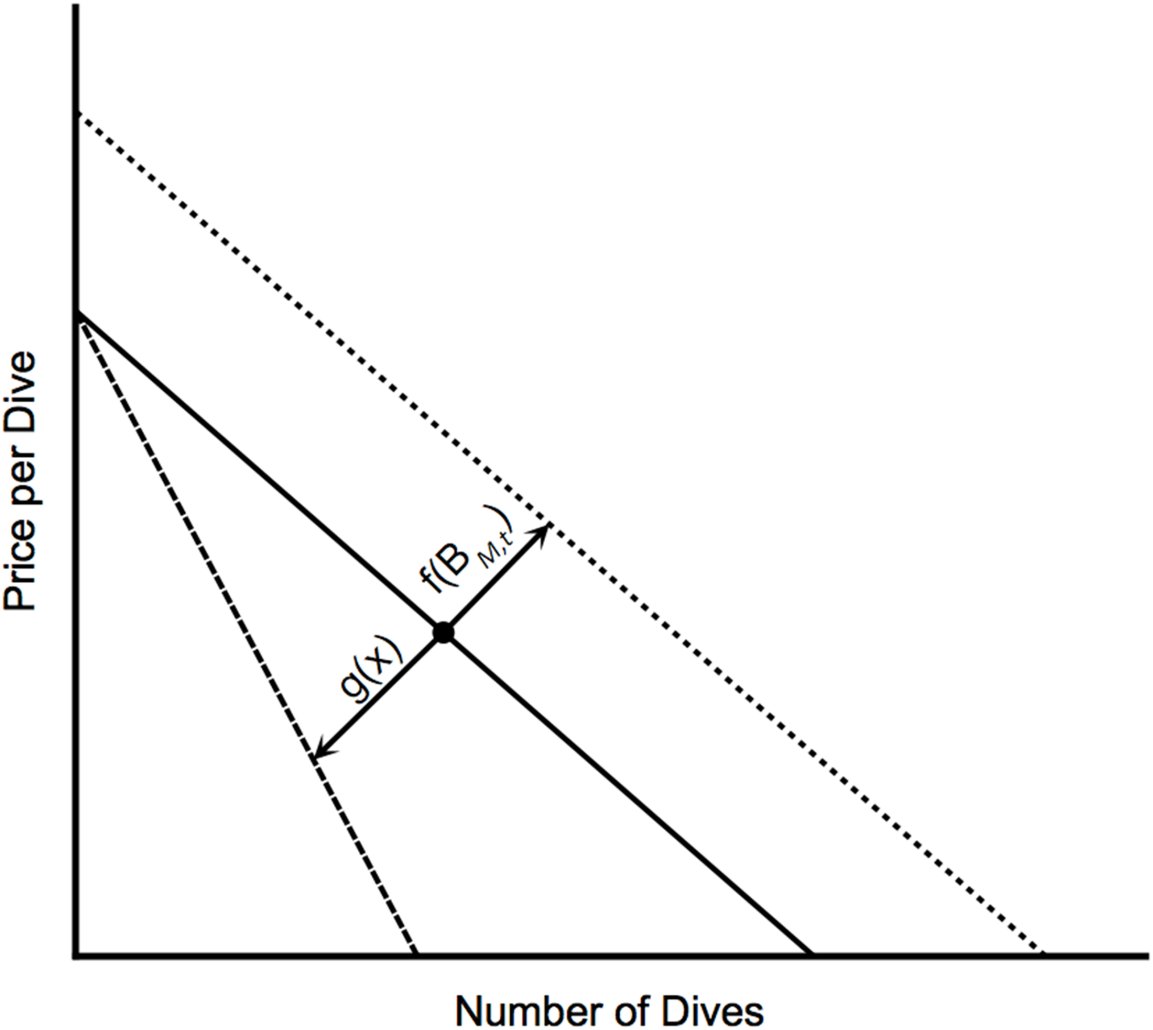
Hypothetical illustration of the effects of congestion, *g(x)*, and fish density, *f(B*_*M*,*t*_*)*, in divers’ demand (Eq 7). Dotted line illustrates Eq 7 at higher fish density levels. Dashed line illustrates Eq 7 at higher congestion levels.

We assume that a subset of patches, denoted by *M*, is designated as a marine reserve. Thus, *f*_*jϵM*_ = 0, and the size of the marine reserve is denoted by *x* = *card*(*M*). Year *t* biomass in reserve is just Σ_*jϵM*_ *B*_*j*,*t*_ which is denoted by *B*_*M*,*t*_. We used a modified version of the equation described by Sala et al. 2013 to model the marginal value of additional dives:

$$P_{t}=\alpha_{0}+f(B_{M,t})-\;{g(x)D}_{t}$$ (7)

where *P*_*t*_ is the marginal value of dive *D*_*r*,*t*_, α_0_ is the intercept of the demand function, *f(B*_*M*,*t*_*)* is the demand shifter reflecting fish abundance in the marine reserve, and *g(x)* changes the slope of demand to reflect congestion of divers in the marine reserve (this congestion effect will depend in reserve size, *x*). The fish abundance effect on demand, *f(B*_*M*,*t*_*)*, is increasing in fish biomass inside the reserve and the congestion effect, *g(x)*, is decreasing in the size of the reserve (Fig 1). The function forms for *f(B*_*M*,*t*_*)* and *g(x)* are given as follows:

$$g(x)=\frac{\alpha_{1}}{{\left({\text{log}}_{100}x\right)}^{\frac{1}{w}}}$$ (8)

where *α*_1_ is a location specific price elasticity, *x* is the reserve size and *w* controls the slope of the logarithmic function. We assumed a logarithmic function because it allows different slopes to be modeled. The different slopes represent distinct levels of tourism potential, reflecting the fact that when there is a high number of possible divers, small reserves cannot capture all potential tourism revenue because of the congestion effect. This allows the model to account for crowding issues and diver carrying capacity, which limits the number of divers per area of reserve. We assume that the diver carrying capacity is set to prevent environmental degradation by divers so that tourism activities does not interfere with biomass buildup inside reserves. By setting a cap on the number of dives, reserve area will directly affect the total revenue that can be generated, especially in locations with high tourism potential (S1 Fig, *w* = 0.25). Under such conditions, tourism value is expected to increase as marine reserve size increases, since more divers will fit in a larger reserve. On the other hand, in locations with low tourism potential, the crowding effect is less important (S1 Fig, *w* = 6). This is expected to happen, because all potential divers can fit in a relatively small area. Thus increasing reserve size does not imply a significant increase in the number of dives. Although maximum tourism values are scaled to one, revenues generated in locations with high tourism potential can be dramatically higher than locations with low tourism potential.

The influence of fish density in the demand curve is represented by *f*(B), which shifts a dive’s marginal value in a logistic manner:

$$f(B_{M,t})=\frac{b}{1+b*e^{-(c\;*\;\frac{B_{M,t}}{K_{M}})}}$$ (9)

Where *B*_*MR*,*t*_ is the total marine reserve biomass, *K*_*MR*_ is the marine reserve carrying capacity, and *b* and *c* are the parameters for the logistic curve that regulate the relationship between density and tourism value. Parameter *b* represents the additional number of dives that can be obtained due to fish density improvements. Parameter *c* regulates the rate of increase and the minimum density level required for tourism value to begin increasing. We assume that the demand for dives in a marine reserve will shift outward through a logistic relationship with fish density. This assumption is meant to address the fact that marine reserves can achieve a certain threshold of fish density where their attraction to divers will grow far more rapidly (at least more than fish density in areas open to fishing) and after a certain point increasing density will attract few additional divers. This relationship is determined by the *c* parameter, with actual values representing different location conditions (S2 Fig). In locations where the main draw to the area is not the marine reserve, fish density may not be as important to achieve a given level of tourism revenues. Under such conditions tourism revenues may start growing even with relatively low fish densities (S2 Fig, *c = 25*). An example of this scenario is Barbados, where divers reported that terrestrial characteristics are the main reason for visiting the area [23]. In addition, such a pool of tourists is likely to have a higher fraction of less experienced divers, for whom the underwater experience is not as important. Conversely, in locations where the main tourism draw is the marine reserve itself, diving experience is more important and dive tourism value will likely increase at higher fish densities (S2 Fig, c = 10). An example of this scenario would be Cabo Pulmo, Mexico, an isolated community where the marine reserve is the primary tourism draw and tourism revenues grew rapidly after a 400% increase in the biomass of targeted species [26].

Eq 7 can be used to calculate the number of dives in a given patch for any given price and biomass level. The optimal price (*OP*_*t*_) that maximizes total revenue can also be calculated by taking the derivative of the product of the fee per dive and the number of dives in the reserve and setting the equation equal to zero:

$${OP}_{t}=\alpha_{0}+\;\left(\frac{\alpha_{0}+f(B_{M,t})}{-2}\right)+\;f(B_{M,t})$$ (10)

Tourism revenue (*TR*_*r*,*t*_) is calculated by multiplying the number of dives in the reserve by the optimal price per dive (*OP*_*t*_):

$${TR}_{r,t}={OP}_{t}\left(\frac{f(B)+\;\alpha_{0}-{OP}_{t}\;}{g(x)}\right)$$ (11)

Equilibrium tourism revenue is calculated as the tourism revenue generated in year 50. Total net present value of tourism revenue (*TV*) is calculated by summing the predicted revenue across all years and applying a discount rate:

$$TV=\sum_{t}^{50}TR_{r,t}*{\left(\frac{1}{1+\delta }\right)}^{t}$$ (12)

Where *δ* is the discount rate.

To obtain general results, we normalize potential tourism and fisheries revenue to each be between 0 and 1, as actual revenue is context dependent. Assuming a 0 to 1 value allows us to test the influence of different relative values from fisheries and tourism on the optimal marine reserve design. Additionally, this assumption does not affect the shape of the tradeoff between these two services, as relative values will only help choose along the tradeoff curve the marine reserve design that provides highest economic returns. We further explore the implication of different relative tourism and fisheries values by demonstrating how actual values can alter optimal marine reserve size. Two metrics are used to determine the value of these services: normalized net present value (NPV) and equilibrium revenue. Net present value of tourism and fisheries services considers the time required for such benefits to be realized. Since future revenues are discounted, timing of benefits becomes a crucial factor. Characteristics such as low initial biomass or slow population growth rates increase the time required for benefits to be realized and therefore negatively affects the NPV. For this metric, a value of one represent the maximum possible NPV that can be achieved for fisheries and tourism services given all possible design and fisheries management options. Equilibrium revenue of fisheries and tourism services does not consider the time component. This would be important for stakeholders that have a long-term vision, without time consideration. For this metric, initial biomass or growth rate are not as important. A value of one represent the normalized maximum equilibrium tourism or fisheries revenue that can be achieved by the system. When considering total revenues, optimal marine reserve design is calculated for different relative values of fisheries and tourism services. Optimal marine reserve size is defined as the design that maximizes total economic value of the system (tourism + fisheries) for every given relative worth of both services. The timing component of the model is also explored more explicitly by calculating the number of years required for particular relative tourism values to be realized under different management scenarios. For default values, we assume a movement fraction (μ) of 0.2, an intrinsic growth rate (r) of 0.2 and a 5% discount rate. For the tourism model we assume a moderate dependence of the revenue on fish density (c = 15) and a moderate crowding effect (w = 1). Sensitivity analysis of all model parameters are shown in the supplementary material.

## Results

**Fig 2:**
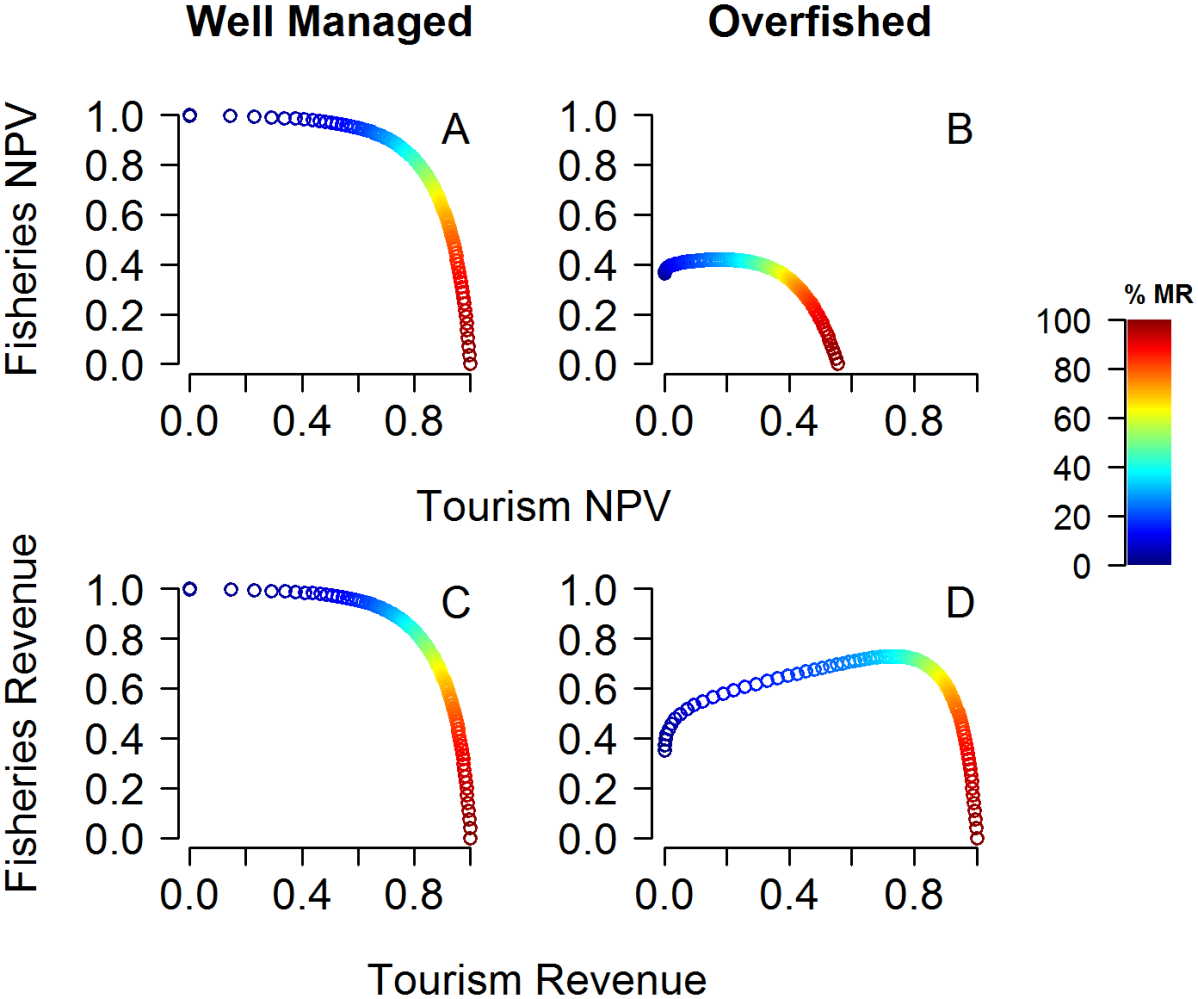
Tradeoffs between fisheries and tourism services for well managed (A and C) and overfished (B and D) scenarios. (A) and (B) demonstrate results in terms of net present value and (C) and (D) demonstrate results in terms of the equilibrium revenue. Colors represent the percent of the area designated as marine reserve.

Expected tradeoffs between fisheries and tourism services vary according to different management scenarios and metrics (Fig 2). In well managed scenarios, maximum fisheries revenue is achieved with no marine reserves. Fisheries revenues decrease as marine reserve size increases. In such cases, if tourism value is ignored, marine reserves are not part of the optimal economic solution. Thus, with perfect fisheries management, accounting for tourism benefits will be crucial for marine reserves to be part of the optimal economic solution. By contrast, in the overfished scenario higher fisheries value can be achieved with marine reserve implementation. Consequently, even if tourism value is ignored, marine reserves will be part of the optimal solution when resources are overfished. When considering the net present value of fisheries and tourism services (Fig 2A and 2B), overfished areas can only obtain a fraction of the total NPV from well managed systems because of the difference in the initial biomass values and harvest levels. In contrast, when considering equilibrium revenues of tourism and fisheries services, overfished scenarios can achieve much higher values relative to well managed systems. This happens, because equilibrium values do not account for the time required for biomass recovery. Thus, since equilibrium values do not consider discount rate, initial biomass is not as important. Additionally, tourism benefits have a maximum value of one in both cases (well managed and overfished), because closing the entire area to fishing does not affect equilibrium values.

Despite inherent tradeoffs between tourism and fisheries services, relatively high values of both services can be achieved simultaneously. For example, for all scenarios where maximum tourism can be achieved (Fig 2A, 2C and 2D), both services can simultaneously achieve about 80% of their maximum value. This is the point along the tradeoff curve that maximizes the sum of both normalized values. Interestingly, when considering equilibrium revenues, a reserve of about 40% is desired to maximize the sum of both values (tourism + fisheries), independent of the management scenario. If for economic or social reasons revenues higher than 80% are desired for one of the two services, it will lead to significant costs to the other. For example, for all scenarios where maximum tourism can be achieved (Fig 2A, 2C and 2D), achieving 90% of tourism benefits will reduce fisheries revenue to about 40% of its maximum value. On the other hand, achieving 90% of fisheries value in well managed scenarios (Fig 2A and 2C) will reduce tourism revenue to about 60% of its maximum value.

Sensitivity analysis of crowding (*w*) and fish density (*c*) effects on the tradeoffs between tourism and fisheries services show that the shape of the tradeoff is sensitive to these parameters (S3 Fig). In locations where diving is not the main driver of tourism benefits (high *c* value), a small marine reserve might be enough to generate the density of fish needed to attract divers. Locations where the marine reserve is the main tourism driver (low *c* value) larger areas are necessary to create the density needed for tourism benefits to be realized. Additionally, strength of the crowding effect will affect the optimal marine reserve design. Since an area can only fit a certain number of divers at any given time, locations with high tourism potential (low *w* value) will require more protection to achieve full benefits. Conversely, in locations with low tourism potential (high *w* value), crowding is not significant. Thus, small marine reserves can accommodate all divers. Simulation of these scenarios shows that although high fish densities can often be achieved with small marine reserves, larger areas may be necessary to capture all potential tourism benefits.

**Fig 3:**
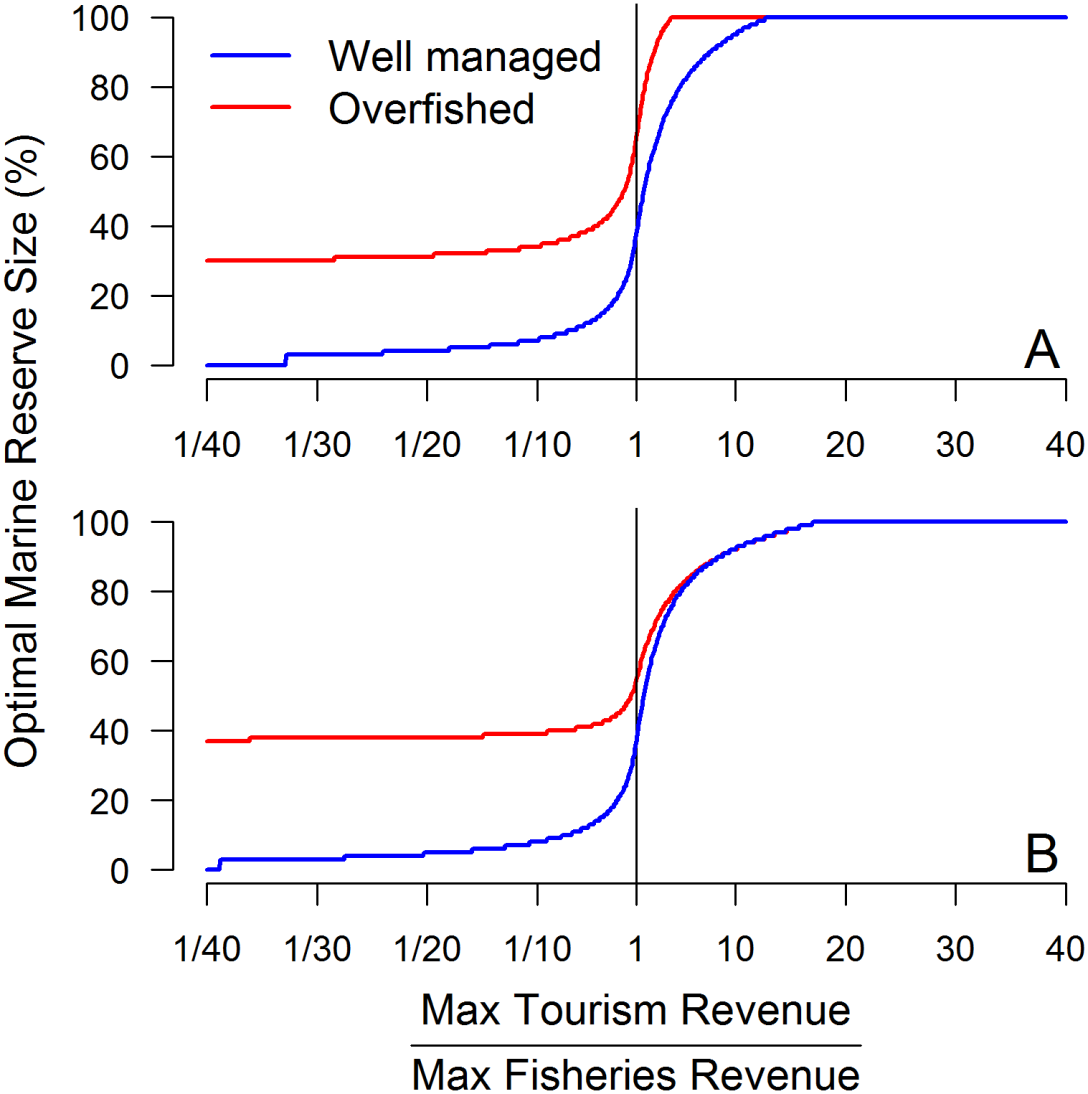
Optimal marine reserve size for different relative net present values of tourism and fisheries services. The two figures represent different outcome metrics, where (A) is in terms of net present value and (B) is in terms of equilibrium revenue.

If the planning objective is to maximize overall revenues from both fisheries and tourism services, actual economic values will be crucial to determine the optimal design. Fig 3 shows the influence of relative tourism and fisheries values on optimal marine reserve size under different fisheries management scenarios and outcome metrics. Generally, optimal marine reserve size increases as relative tourism value rises, eventually reaching 100% of the area. For overfished scenarios, marine reserves are always part of the optimal solution, even with relatively low tourism values. The optimal marine reserve size for overfished scenarios when tourism value is extremely low is about 30% of the area. As the relative revenues from tourism and fisheries reach a value close to one, optimal marine reserve size increases rapidly, eventually reaching 100% of the area (Fig 3). In well managed situations, where marine reserves are not part of the optimal solution for fisheries alone, including tourism value changes the outcome even when tourism revenues are well below fisheries values. This happens, because even tiny reserves (1–2%) can bring larger tourism value than the corresponding losses to fisheries value. As tourism value increases relative to fisheries, the optimal marine reserve size grows, eventually reaching 100% of the area. When considering equilibrium revenues (Fig 3B), optimal marine reserve area is very similar for both management scenarios when tourism value is about 10 times fisheries value. Sensitivity analysis to crowding and fish density effects show that optimal marine reserve size for different relative values of tourism and fisheries services can be quite different depending on these parameters values (S4 Fig). Generally, as tourism potential and crowding effect decreases (high *w* values), higher relative tourism value is needed for high levels of protection. Since higher relative tourism versus fisheries values are harder to achieve when there is low tourism potential, closing big portions of the area becomes less likely. Additionally, with low tourism potential, fish density effect plays an important role. As dependence on fish density increases (locations where the main draw is the marine reserve) greater protection is desired.

**Fig 4:**
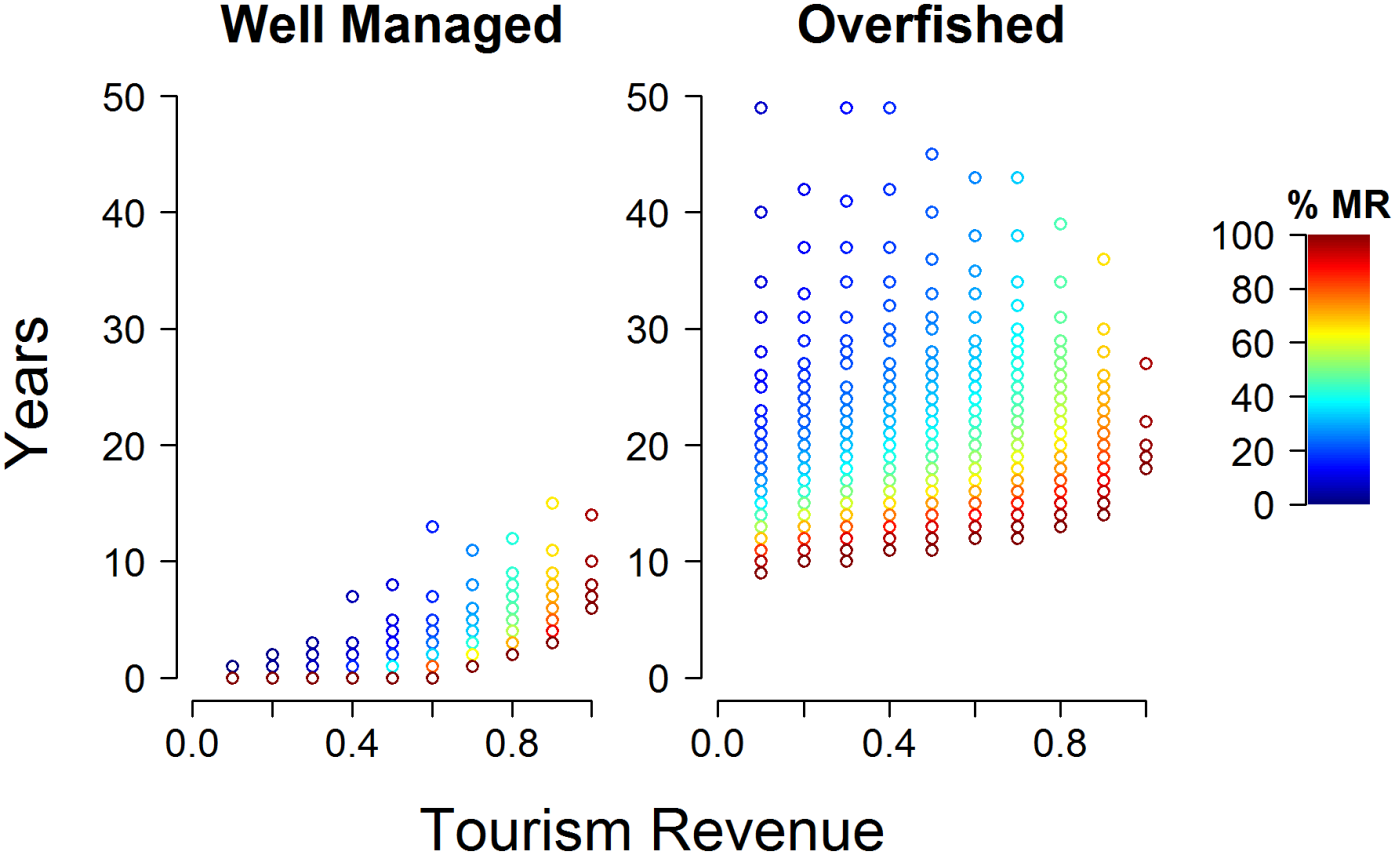
Timing of tourism revenue for different marine reserve sizes and fisheries management scenarios.

Timing of benefits is an important factor to consider when creating a marine reserve expecting tourism gains. Fig 4 demonstrates the number of years required for tourism revenues to be generated under different marine reserve designs. Because of different starting points and intensities of fishing in open areas, the timing of benefits varies significantly. In well managed scenarios tourism benefits can happen relatively quickly, because stocks inside the marine reserve start at higher values. By contrast, overfished scenarios can take much longer for marine reserve densities to reach peak values. Additionally, the larger the area protected the quicker benefits will be realized, because fewer fish leave the boundaries of the reserve where they can be caught. For example, it takes 40 years to achieve 0.5 of the maximum tourism revenue in overfished scenarios for a marine reserve size of 25%. The more tourism revenue is dependent on fish density the longer these benefits take to be realized (S5 and S6 Figs).

The benefits of reserves are sensitive to growth and movement characteristics of the target species (S7 Fig). Generally, species that have high movement rates and low growth rates will require larger reserves to achieve tourism benefits. This is consistent with the literature on biological responses of marine reserves, where species that move more require larger areas to be protected [10]. On the other hand, if movement rate is close to zero, relatively small areas will be sufficient to produce fish densities that attract divers, and tourism value will depend mostly on the strength of the crowding effect. Additionally, effects of growth and movement rates on optimal design is greater in overfished relative to well managed scenarios because of the difference in fishing mortality of the fish that spill over from reserves.

## Discussion

Accounting for tourism benefits can significantly influence optimal marine reserve design. Results from our model show how considering tourism objectives can be crucial for marine reserves to be part of the optimal economic solution, regardless of the state of the fishery. This result challenges previous findings that marine reserves are not part of the optimal economic solution when the fishery is well managed [31]. Our findings demonstrate how marine reserves should be implemented even in situations with optimal fisheries management and low tourism value relative to fisheries. In such situations, a relatively small reserve can generate more benefits from spillover and tourism development than foregone fisheries value. As tourism value increases relative to fisheries revenues, larger areas should be protected to maximize economic outcomes.

Conflicts are likely to be highest when there is clear preference for one service over the other. Optimal marine reserve design choice will be greatly influenced by the relative social and economic value of tourism and fisheries services. Different stakeholders can have distinct social tradeoffs, which can be related to higher relative profits, social motives such as employment, or cultural reasons such as local traditions and customs. Therefore, some stakeholders might care more about one service than the other, influencing the optimal marine reserve design and inherent tradeoffs between fisheries and tourism services. Stakeholders that depend solely on resource extraction, such as fishers, might only value fisheries and not care about tourism benefits. Consequently, optimal design will be the point along the tradeoff curve that maximizes fisheries value—i.e., the location where a horizontal line reflecting a pure preference for fishing is tangent to the tradeoff curve. Generally, greater preferences for fisheries services will lead to lower tourism values and less area being protected. In well managed scenarios, the optimal solution that maximize fisheries services is to open the entire area to fishing and manage the fishery well. This will lead to zero tourism value, since our model assumes that revenue is generated through the collection of user fees that depend on marine reserve establishment. In overfished scenarios, marine reserves are required to maximize fisheries value. This creates a win-win situation with tourism services, where maximizing the value of one service also generates value to the other service. Stakeholders that rely only on tourism activities (e.g dive operators) might have a high preference for tourism benefits and may not care about fisheries services. When there is a pure preference for tourism services optimal marine reserve design is where tourism value is maximized. This happens where a vertical line is tangent to the tradeoff curve, which for all scenarios is where 100% of the area is set as marine reserve. The greater the preference for tourism services the bigger the compromise to fisheries revenue, leading to a strong tradeoff between the two services. Stakeholders that value both services equally for economic or social reasons (e.g., government managers) might not have a preference for one or the other service. Thus, following general economic theory, optimal design will be the point along the tradeoff curve that maximizes the sum of both relative values. In this case, optimal design is where a 45 degree line is tangent to the tradeoff curve. For all scenarios, this equal weighting point has relatively high values of both services and relatively low tradeoffs.

Revenues generated through user fees can be used in many ways to offset potential costs associated with the marine reserve. Revenues can be used for direct compensation to fishers, investment in better management of fisheries, creation of alternative livelihoods, community infrastructure, and/or monitoring and enforcement. Direct compensation to fishers can be used to compensate for losses associated with reduced fishing grounds. One example of such a scheme is in the Great Barrier Reef Marine Park, where the Australian government provided compensation for commercial fishers adversely affected by the reserves [32]. Such schemes might be useful to obtain support from key stakeholders, but can create perverse incentives to overharvest areas that are still open to fishing. Utilizing revenues to invest in better management for adjacent areas open to fisheries can be an alternative to achieve long term sustainability of the fisheries. Although such investment would not address short term costs, it can help to ensure spillover benefits from marine reserves to affected fishers and achieve better fisheries profits in the future. For example, in the Galapagos National Park, revenue from fees are used by the government to manage the fisheries around the islands [33]. An alternative to investing in the fisheries sector would be to invest in alternative livelihoods such as aquaculture or tourism. Such alternatives can have many positive effects by increasing resilience of the system through income diversification. Additionally, it can decrease problems associated with the displacement of effort to outside areas by converting some of those fishers into tourism operators or aquaculture farmers. For example, in the Raja Ampat Marine Reserve system located in Indonesia, 30% of the user fees are directed to communities in the region for projects related to tourism development [13]. The remaining revenue is used for managing the marine reserves, including costs related with monitoring and enforcement. In many cases, benefits generated through user fees are entirely used by government agencies or NGOs for monitoring and enforcement of the area [34,35]. Using all revenue for reserve management can help enforcement of the area but does not address the root of the problem. In such cases, fishers typically bear all costs and do not have any secure benefits from the reserves, which can lead to strong opposition to any marine reserve creation. Therefore, uncertainty and timing of fisheries benefits might lead to increased illegal activities and enforcement costs, which is one reason for many “paper parks” worldwide [5].

Short term costs to fisheries [36] and long term maintenance costs of marine reserves can be a strong deterrent to their success [5]. As fisheries and tourism benefits are related to the density of fish inside closed areas, such benefits can take a long time to be realized depending on reserve size, species characteristics and the fishing pressure before and after reserve creation [37]. Results from our model demonstrate how tourism revenues generated through user fees can take many years to be realized, especially when the fishery is overfished prior to reserve creation. With such benefits occurring in the future, innovative market strategies might be needed to compensate for short term fisheries losses. Such market-based strategies can be a promising solution to use future tourism benefits to offset short-term fisheries losses [36]. For example, in areas with high tourism potential, significant revenues are expected in the future. Thus, agreements between the tourism industry and fishers can be established to ensure fishers are guaranteed a share of future tourism benefits. Although this alternative does not address short term losses it ensures future benefits to fishers, which might be sufficient to gain their support. The level of support might in turn depend on the timing of such benefits and discount rate of the fishers. If their discount rate is high, future benefits can be insignificant compared to short term losses. Another market-based alternative might be to acquire a loan with banks or philanthropic organizations to compensate short-term losses, with payments from future tourism benefits. Philanthropic organizations interested in marine conservation might offer lower discount rates than banks and are usually more willing to take the risks related to an uncertain benefit. The magnitude of uncertainty on future tourism benefits will likely depend on the characteristics of the area related to their tourism potential. For example, in areas where there are no other major attractions other than the marine reserve, marketing campaigns and tourism infrastructure need to be fomented to create a reputation of the area among the diving community and provide minimal conditions for tourists. Otherwise, there is a chance that tourism benefits are going to take too long or will not happen at all, especially if diving experience is not spectacular enough to compete with other marine reserves from around the world.

Our model assumes that tourism revenue is associated with the density of fish inside the marine reserve. Although fish density is one of the main ecosystem attributes preferred by divers [19], other characteristics can also be important. For example, diversity and size of fish and corals can be an important factor for divers [20]. Although we don’t explicitly account for these characteristics, such attributes are generally correlated with increases in density inside marine reserves [38]. Additionally, we assumed that divers are driven by only one species of fish, while in reality there will undoubtedly be far more than one important species. Optimal marine reserve design will vary depending on the biological characteristics of the species and focusing on only one may not be sufficient to increase the biomass of the other. One approach would be to focus on the species that have the greatest movement rates to ensure positive growth of all species. Focusing on species with high mobility would mean having to close a relatively large area, which might be challenging depending on the context. For example, locations with low tourism potential and high tradeoffs with fisheries services, protecting large areas might not be viable. On the other hand, for locations with high tourism potential that depend on mobile species for diving activities, benefits from protecting a large area likely outweighs potential costs to the fisheries sector. Another important assumption of our model is that fisheries target the same species that divers care about. In cases where the main draw for divers are charismatic species not targeted by fisheries (e.g. dolphins, whales, turtles), such an assumption might not hold true. Although such species are not expected to be directly affected by protection as much as species targeted by the fisheries, marine reserves can provide indirect benefits through increased food availability [39]. Additionally, even though there might not be any significant increases in density, marine reserves still create an instrument to collect revenue that can be invested in the region.

Tourism activities inside marine reserves can have positive and negative effects for marine conservation. Since tourism activities are dependent on marine conservation, high synergies between tourism and conservation services can be expected. Additionally, having a regular presence of divers in the reserves can help with monitoring and enforcement of the area as it can discourage poachers and facilitate detection of illegal fishing activities. On the other hand, inexperienced divers can cause significant habitat degradation and alter important fish behaviors [40]. Many studies have pointed out the damage caused by divers in sensitive coral reef areas [41,42]. Prevention of damage can be achieved by setting a maximum number of divers for a given area [43] and providing proper training and education to dive masters and recreational divers about best practices and potential harms associated with this activity. Several marine reserves around the world have been using a diving carrying capacity to minimize environmental damage caused by divers. For example, the Mendes Islands Marine Reserve has established a maximum of 450 dives per day [16]. Protecting large portions of the ocean can also help decrease diver density and increase potential conservation benefits. Such methods can significantly decrease adverse tourism effects and increase synergies between conservation and tourism services. In our model, we assume that a maximum number of dives per reserve area is set to prevent environmental degradation by divers. Thus, diving activities does not interfere with biomass buildup inside reserves. Future research can relax that assumption and explore how environmental impacts by divers interfere with design outcomes.

We use a conservative model in terms of the benefits that can be generated to fisheries. First, our model only considers adult spillover as benefit source. It does not account for potential recruitment increases through larval and egg spillover which in many cases can be the main source of benefit [44,45]. We did not include larval dispersal dynamics in our model in order to obtain simplified but conservative results. In our model, when adult movement rate is zero, there is no possible source of benefit to the fishery. This is not true in many cases where marine reserves can be an important source of eggs and larvae to fished areas thus increasing recruitment and growth rate of the fished population. This can have important design implications in terms of reserve location and the expected recruitment benefits to fished areas [27]. Second, we assume that effort is going to remain constant through time, being redistributed into fished areas after reserve creation [46]. This causes an increase in fishing mortality in the outside areas as marine reserve increases and the fishing the line effect [29]. This assumption can be true in many situations with weak management outside reserve boundaries. If the fisheries are optimally managed, fishing effort is expected to adjust in order to provide optimal economic returns. As marine reserves increase, overall effort in outside areas should be decreased and concentrated near reserve borders to optimize economic returns [8,46]. Effort reduction can be facilitated with increased tourism activities as it can create alternative livelihoods for the local community. Thus, even though we conservatively assumed that effort is going to remain constant, tourism activities in the reserve might in reality decrease fishing effort in outside areas. For example, in Raja Ampat—Indonesia, many locals that used to depend on fishing as their main source of revenue are transitioning to the tourism sector using user fee revenues to invest in local tourism infrastructure [13]. Additionally, increased tourism activities might influence local consumption of sustainable seafood and increase the price of locally harvested products, allowing reductions in catch without compromising total revenue generated.

## Conclusion

Our model provides the first attempt to incorporate future tourism revenue in the design of marine reserves. Tourism is a way to capture benefits from conservation and turn it to a monetary value, which is crucial when comparing with fisheries value. We provide significant insights on the importance of the specific location characteristics in the prediction of future tourism benefits. Previous tourism infrastructure and other local attractions can play a critical role in determining the expected benefits and their relationship with fish density. Tourism potential of each area can also have significant implications to marine reserve design because of congestion effects. In all scenarios tested, marine reserves were part of the optimal design when considering both tourism and fisheries benefits, even when the fishery is well managed outside. The amount of area to be protected will greatly depend on the value of tourism relative to fisheries. As relative tourism value increases, the percent of the total area to be protected also increases. In areas where tourism value is orders of magnitude greater then fisheries value, it would be optimal to close the entire area to fishing. Therefore, accounting for tourism benefits can be crucial to optimally design marine reserves. Additionally, the use of revenues generated through user fees to offset potential costs associated with reserve creation can be crucial to gain support of local stakeholders and increase conservation effectiveness.

## Supporting information

S1 Fig Relationship between congestion effect, *f(x)*, and marine reserve size for different tourism potential scenarios (represented by *w*). (TIFF)

S2 Fig Relationship between the effect of biomass on potential tourism value, *f(B)*, and fish density inside the marine reserve. Different *c* values represent distinct location characteristics.
(TIFF)

S3 Fig Sensitivity analysis of the tradeoffs between fisheries and tourism services for well managed and overfished scenarios under different crowding (w) and fish density (*b*) effects. (TIFF)

S4 Fig Sensitivity analysis of the optimal marine reserve size for different relative values of tourism and fisheries services under different fisheries management scenarios, and fish density (*b*) and crowding effects (*w*) on tourism net present value. (TIFF)

S5 Fig Sensitivity analysis of timing of tourism revenue for different marine reserve sizes to crowding effects (*w*) and fish density effect (*c*) for well managed scenarios. (TIFF)

S6 Fig Sensitivity analysis of timing of tourism revenue for different marine reserve sizes to crowding effects (*w*) and fish density effect (*c*) for well managed scenarios. (TIFF)

S7 Fig Sensitivity of the optimal marine reserve size to growth and movement rates in a 1:1 relative fisheries and tourism value for overfished and well managed scenarios. (TIFF)
