# Correction: Accounting for tourism benefits in marine reserve design

**DOI:** 10.1371/journal.pone.0192127

**Published:** 2018-01-25

**Authors:** The PLOS ONE Staff

The Data Availability statement is incorrect. The correct Data Availability statement is: All results presented in the manuscript were produced from model simulations. Therefore, there is no data to be made available. Researchers who wish to replicate the study will use the equations and parameters described in the manuscript. With such equations and parameters, researchers can use modeling simulations to replicate the figures presented in the manuscript. The publisher apologizes for the error.
