# Supplementary figures and images for: Accounting for tourism benefits in marine reserve design
Source: PLoS One. 2017 Dec 21;12(12):e0190187. doi: 10.1371/journal.pone.0190187 (PMC5739484; doi:10.1371/journal.pone.0190187)

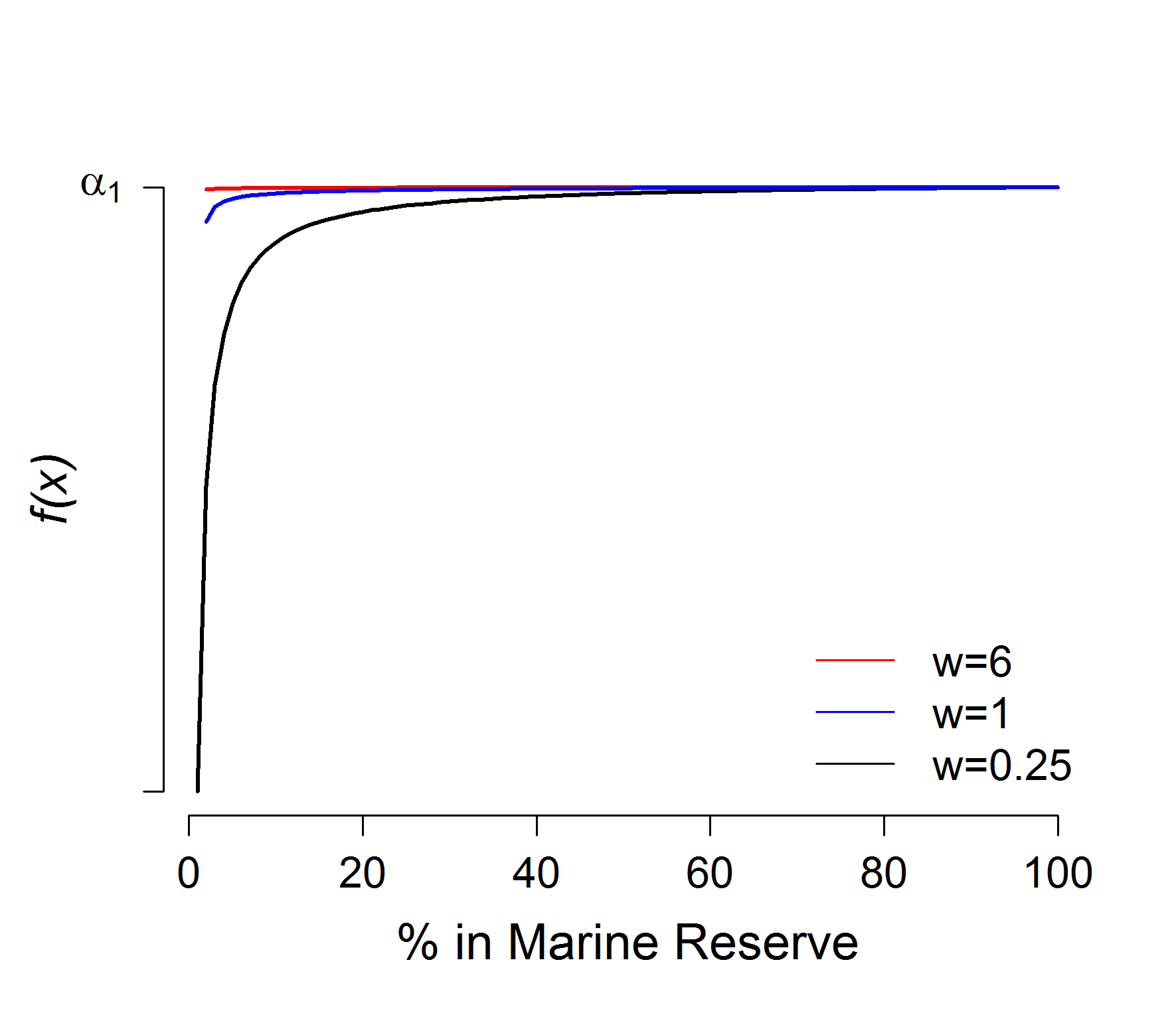

Supplement: S1 Fig — (TIFF) [file pone.0190187.s001.tiff]

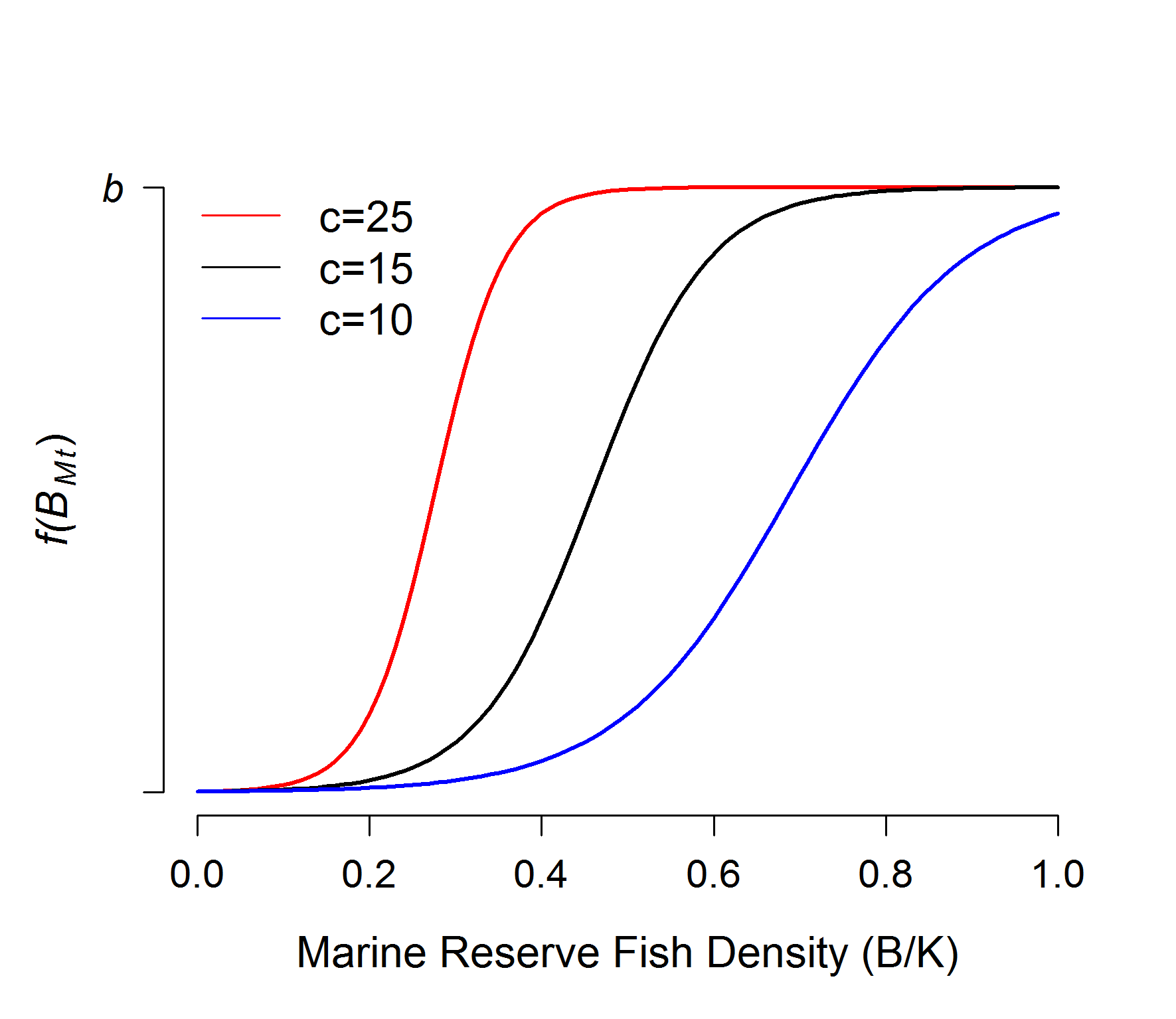

Supplement: S2 Fig — Different c values represent distinct location characteristics. (TIFF) [file pone.0190187.s002.tiff]

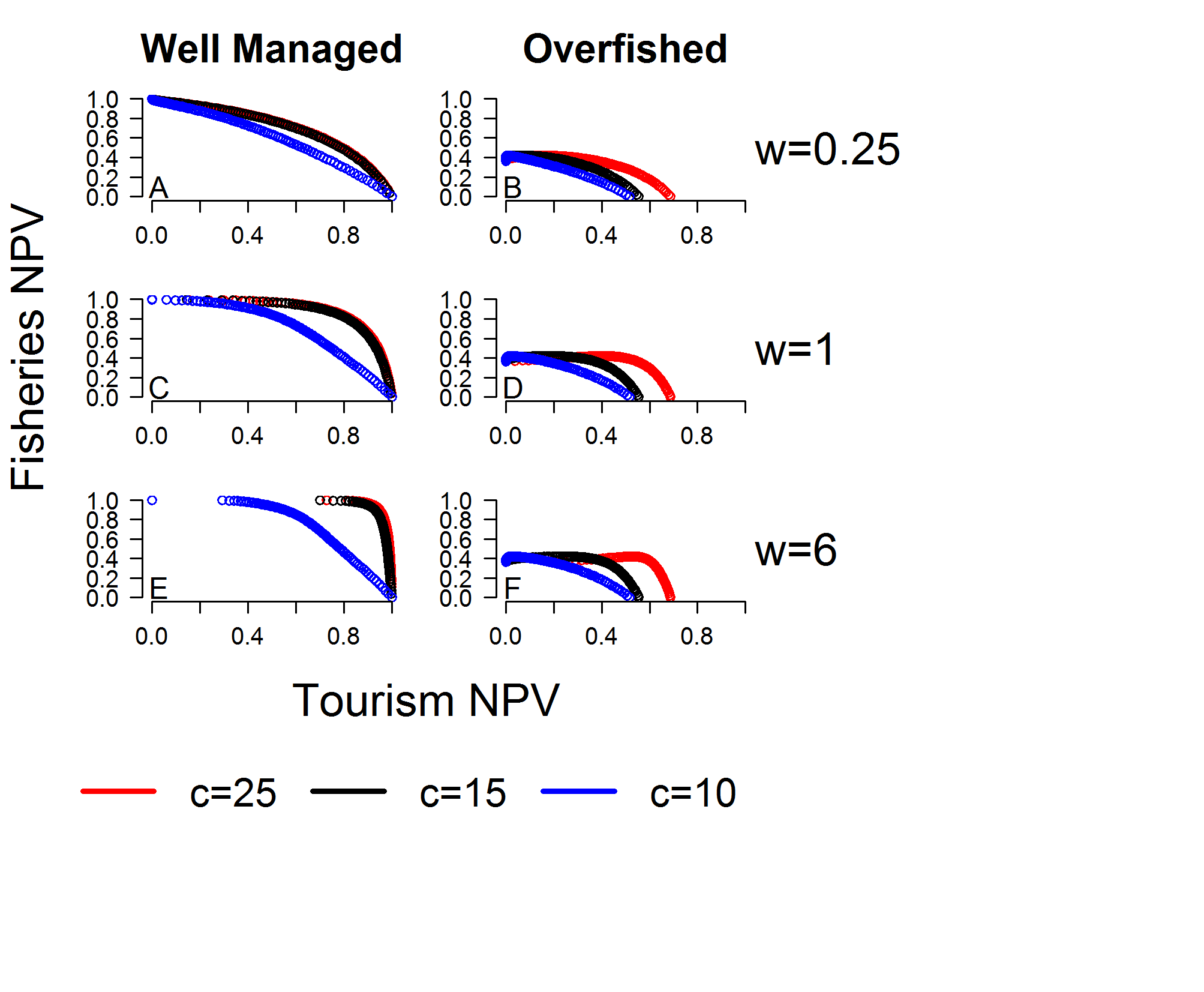

Supplement: S3 Fig — (TIFF) [file pone.0190187.s003.tiff]

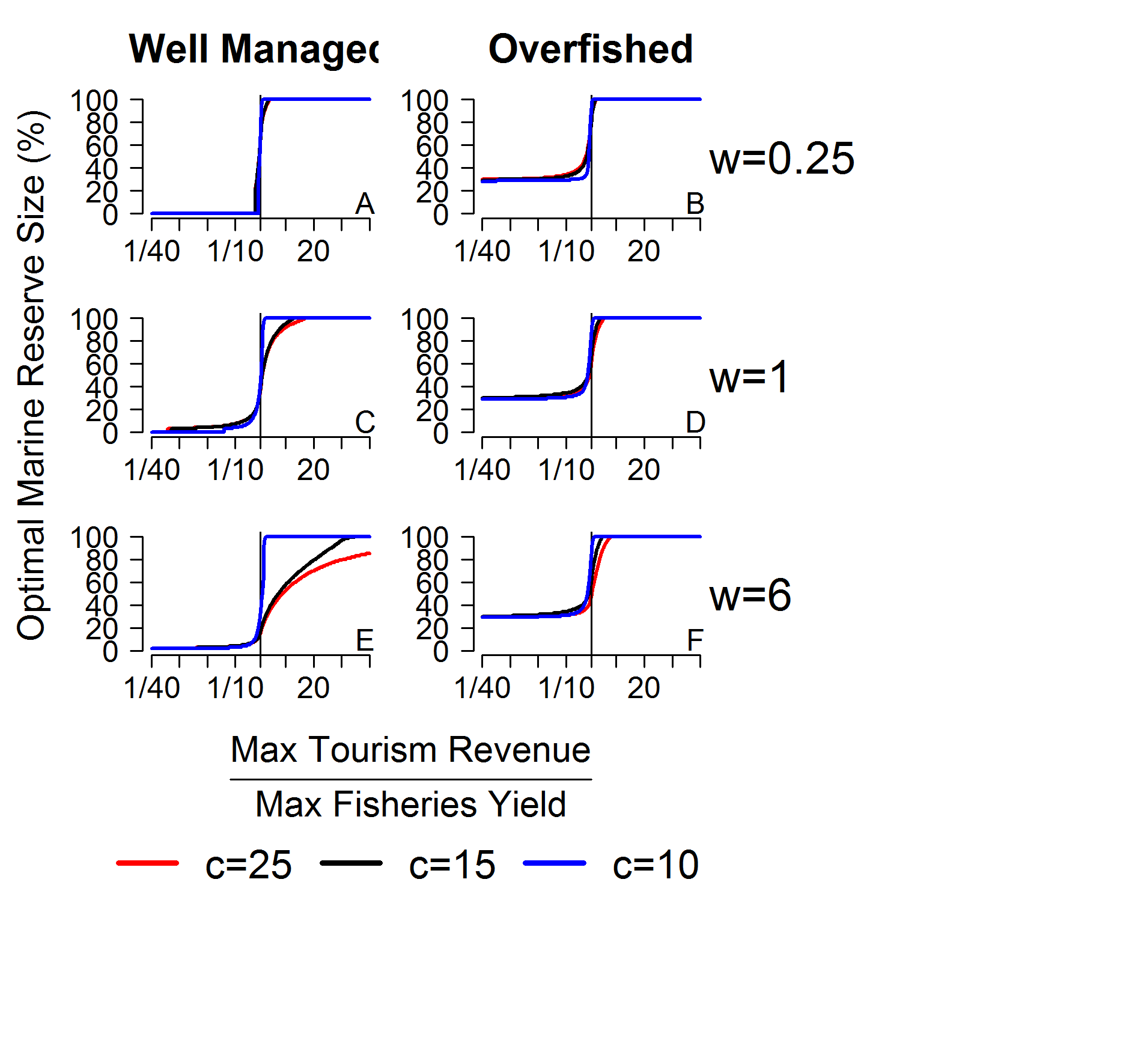

Supplement: S4 Fig — (TIFF) [file pone.0190187.s004.tiff]

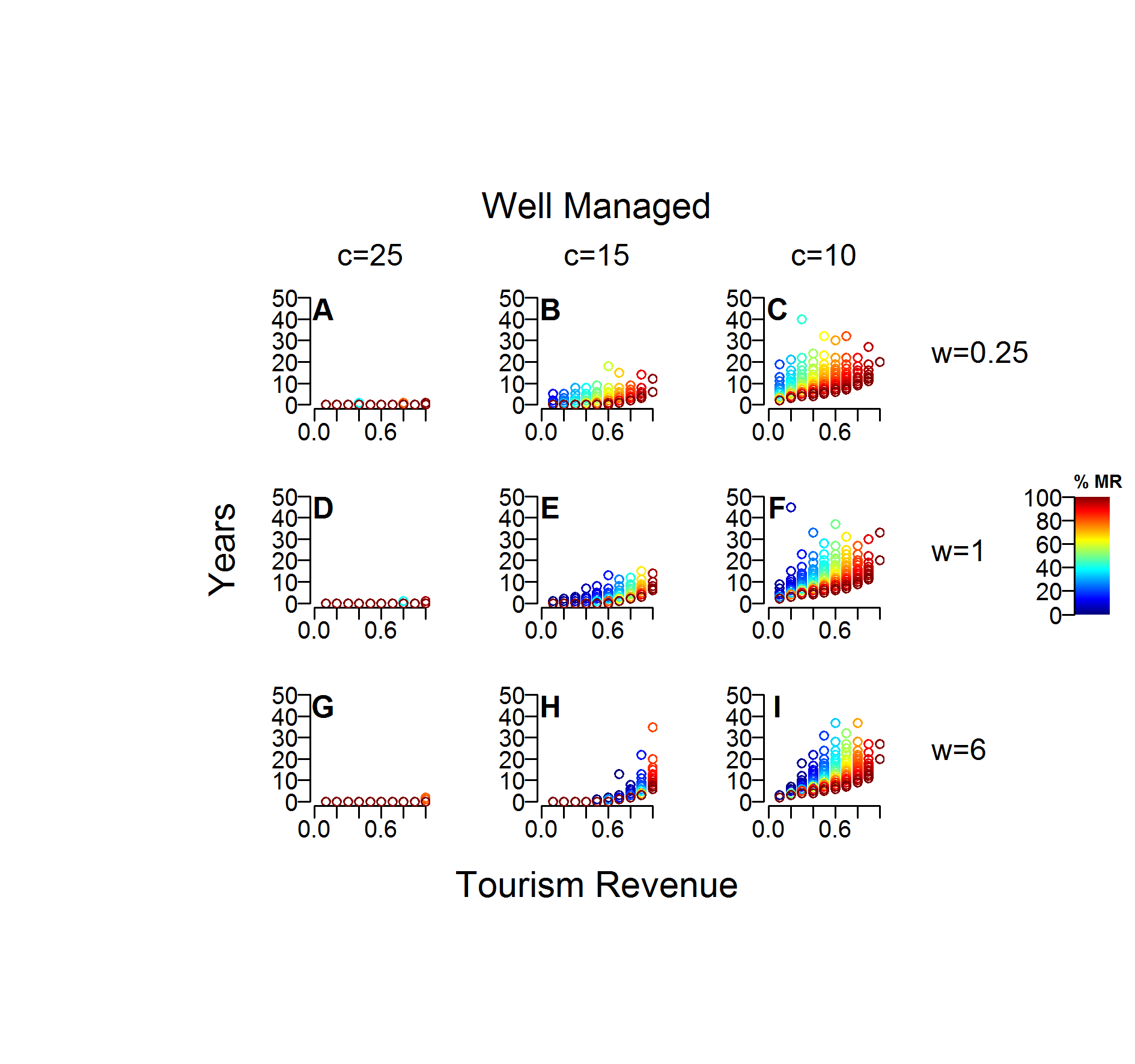

Supplement: S5 Fig — (TIFF) [file pone.0190187.s005.tiff]

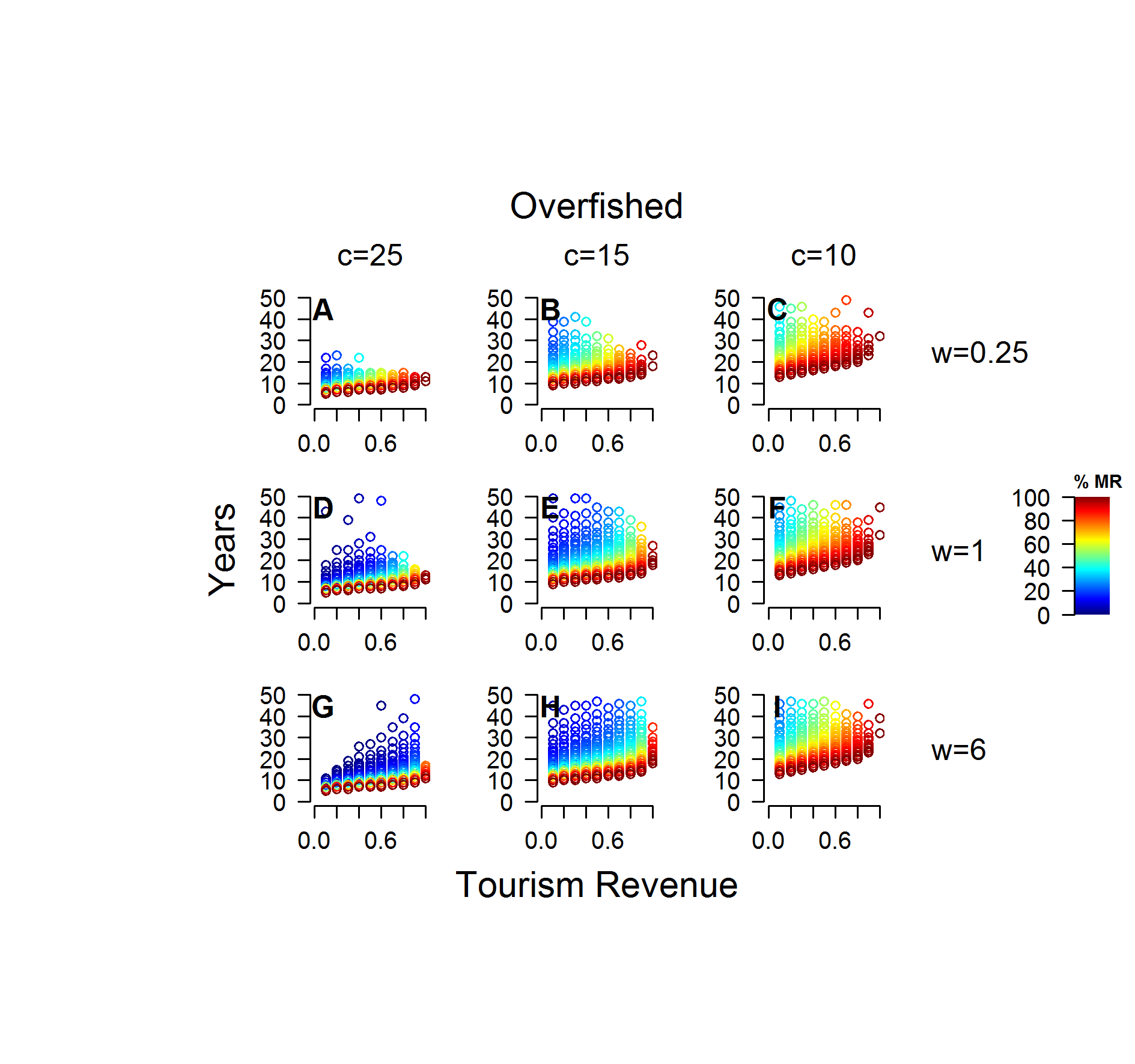

Supplement: S6 Fig — (TIFF) [file pone.0190187.s006.tiff]

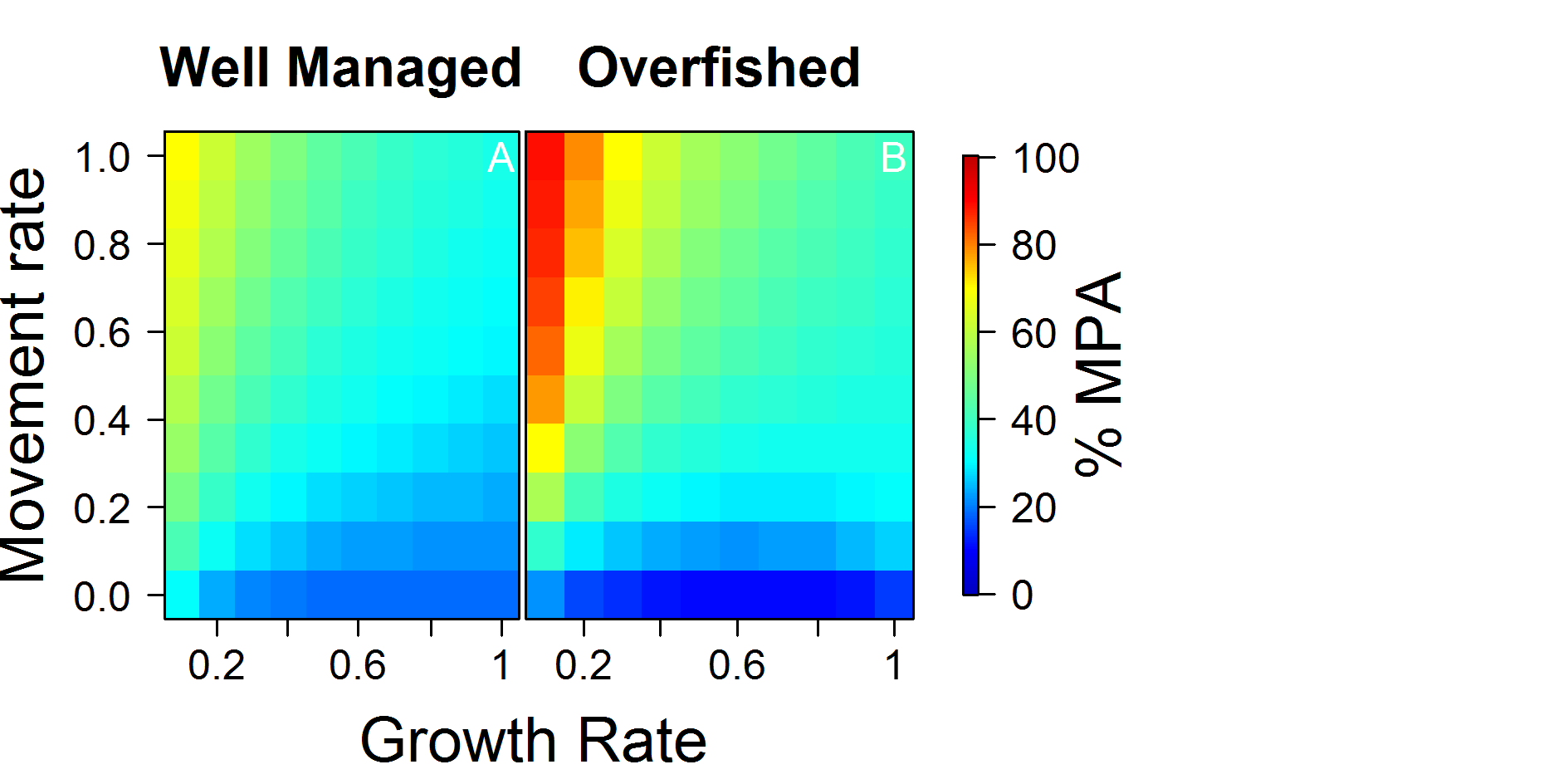

Supplement: S7 Fig — (TIFF) [file pone.0190187.s007.tiff]
